# Application of the Respondent-Driven Sampling method in an online survey in Brazil: COVID-19 morbidity in the post-pandemic period

**DOI:** 10.1590/1980-549720260004

**Published:** 2026-01-30

**Authors:** Paulo Roberto Borges de Souza, Giseli Nogueira Damacena, Euclides Ayres de Castilho, Wanessa da Silva Almeida, Crizian Saar Gomes, Deborah Carvalho Malta, Célia Landmann Szwarcwald

**Affiliations:** I Fundação Oswaldo Cruz, Institute of Scientific and Technological Communication and Information in Health, Laboratory of Information in Health – Rio de Janeiro (RJ), Brazil.; II Universidade de São Paulo, School of Medicine, Department of Preventive Medicine – São Paulo (SP), Brazil.; III Universidade Federal de Minas Gerais, School of Nursing – Belo Horizonte (MG), Brazil.

**Keywords:** COVID-19, Survey, Respondent-driven sampling, Post-stratification, Sociodemographic factors

## Abstract

**Objective:**

To describe the methodological procedures used to collect and analyze data from an online survey carried out from July to December 2023 and to verify whether the obtained sample is representative of the Brazilian population.

**Methods:**

A cross-sectional epidemiological study was carried out through social media, using the Respondent-Driven Sampling methodology to collect information from the Brazilian adult population. For sample weighting, a post-stratification procedure was used based on sociodemographic data of the 2022 Continuous National Household Sample Survey (PNADC-2022). Prevalence estimates of chronic noncommunicable diseases, self-rated health, and health behaviors estimated in the ConVid-2 survey were compared with the estimates from the 2019 National Health Survey (PNS-2019).

**Results:**

A total of 3,805 individuals participated in the ConVid-2 survey. When comparing sociodemographic variables from ConVid-2 with those from the PNADC, we verified very similar proportions in both surveys. Regarding chronic noncommunicable diseases, we found discrepancies only in prevalence of asthma and depression in comparison with PNS-2019. As for lifestyle habits, the percentages of current smoking, adequate leisure-time physical activity, and consumption of healthy and unhealthy foods were similar in both surveys. The greatest difference was observed in screen use after the COVID-19 pandemic.

**Conclusion:**

The use of the Respondent-Driven Sampling method with the inclusion of recruiter-recruitee pairs enabled us to obtain more reliable estimates. Concerning lifestyle changes and the findings of this study, we highlight the need for actions aimed at promoting healthy behaviors and achieving greater advances in the control of chronic noncommunicable diseases.

## INTRODUCTION

The great expansion of the Internet along with the growth and improvement of information technologies has made distance communication much more comprehensive and accessible to health information^1,2^.

In Brazil, in 2024, according to data from the Brazilian Institute of Geography and Statistics (IBGE), 93.6% of permanent private households used the Internet^3^. Faced with this quite universal access, there have been significant changes in the way Brazilians communicate, obtain, and share information, composing a huge network of connected people, regardless of their geographical location^4,5^.

The restrictions imposed by the coronavirus pandemic limited the performance of face-to-face research. Technological advances in the obtainment and sharing of health information and the need to expand knowledge of COVID-19 have boosted the use of online surveys in several countries^6^. In addition to adherence to protective measures and restriction of physical contact^7^, researchers addressed other issues related to the pandemic period such as symptoms^8,9^, psychological disorders^10,11^, difficulties in access to health services^12^, and lifestyle changes^13^.

In Brazil, in 2020, in order to investigate changes in lifestyle and health conditions of the Brazilian population during the COVID-19 pandemic, the *ConVid – Pesquisa de Comportamentos* (ConVid – Behavior Survey) was conducted online^14^. This survey used a snowball sampling procedure to collect information^15^. To adapt the method to be used online, the recruitment process began with the access link to an electronic questionnaire, which was sent by email or some social media. Participants received a request to share their participation invitation with their contact network^16^.

According to the results of the ConVid survey conducted with the Brazilian adult population, there were significant losses in terms of work and income, accentuating social inequalities^17^. There was worsening in self-rated health^18^, sleep and psychological disorders^19,20^, and lifestyle changes. There was an increase in physical inactivity, excess screen time (cell phones, tablets, computers), smoking habit, and alcohol use^21,22^.

In 2023, to investigate issues related to morbidity by COVID-19 in the post-pandemic period, a new survey was conducted, called *ConVid-2 — Pesquisa de Comportamentos* (ConVid2 – Behavior Survey). An electronic self-reported questionnaire was used, filled by the participant by cell phone or computer with access to the Internet. In this second stage of the research, the sampling methodology was improved. The recruitment was carried out by the Response-Driven Sampling (RDS) method^23^, which began by sending a fixed number of invitations with the access link to the electronic questionnaire.

In this article, our objectives are to describe the methodological procedures used for data collection and analysis in the ConVid-2 survey and to verify whether the obtained sample is representative of the Brazilian population.

## METHODS

### Study design

This is a cross-sectional epidemiological study conducted through social media, using the RDS methodology for the collection of information on the Brazilian adult population (aged 18 years or over) from July to December 2023.

The ConVid2 – Behavior Survey project was approved by the National Commission of Ethics in Research (*Comissão Nacional de Ética em Pesquisa* – CONEP) on December 22, 2022, under protocol No. 5.836.202.

The research was carried out by Fundação Oswaldo Cruz, in partnership with the following Brazilian universities: Universidade Federal de Minas Gerais, Universidade Estadual de Campinas, Universidade Federal de Ouro Preto, and Universidade Federal de Sergipe.

People aged 18 years or over, living in Brazil during the COVID-19 pandemic, participated in the study.

### Respondent-Driven Sampling method

The method chosen for recruiting individuals was the RDS, proposed by Douglas Heckathorn in 1997^23^. This is a variant of the snowball sampling method, in which members of the population group under study recruit their peers to participate.

When applying the RDS method, data is collected by successive recruitment cycles, called "waves." First, individuals of the target population, called "seeds," are selected in a nonrandom way to participate in the study, thus starting the recruitment process. Seeds are requested to recruit a fixed number of individuals among their acquaintances from the same population subgroup. The connections of the recruiter-recruitee pairs are recorded based on codes assigned to each participant^24^.

In the recruitment carried out by the RDS method, the recruitment memory is formed wave by wave, following a Markov process. In other words, the characteristics of recruitees depend only on the characteristics of their recruiters, and not on those of the ones who recruited their recruiters or participants of previous waves. After a sufficient number of waves, the characteristics of the individuals in the final sample become independent of those of the initial sample^24^.

The tendency of a participant to recruit individuals with similar characteristics is called "homophily effect."^25^ As the registration of the connections of the recruiter-recruitee pairs is recommended in the RDS method, the bias related to the nonrandom selection of individuals is taken into account as well as the possible overrepresentation of certain characteristics in the population^25^. Thus, RDS can be considered a complex sampling method, with unequal selection probabilities and clusters composed of people recruited by the same participants. To estimate the variances of the indicators of interest, the bootstrap procedure is used through several simulations of samples generated with the same process that originated the total sample^26^.

### Sample size

Assuming a simple random sample, the minimum sample size required to estimate prevalence values of 5%, with confidence intervals of 95%, and two-tailed error of 1% is approximately 1,800 people. As this is peer recruitment, a design effect of 2 was considered and the minimum sample size was calculated at 3,600 people^24^.

### Sample weighting

To obtain a representative sample of the population according to the sociodemographic characteristics of the Brazilian population aged 18 years or over, a post-stratification procedure was used^16^. The considered variables were: sex (men; women), age group (18–39; 40–59; 60+ years), level of education (some high school or lower; high school or some college; college degree or higher) and race/skin color (white; nonwhite). The post-stratification procedure was based on the population estimates of the IBGE's 2022 Continuous National Household Sample Survey (*Pesquisa Nacional por Amostra de Domicílios Contínua* – PNADC-2022)^3^.

### Information collection

To begin the stage of information collection by RDS, supporters (seeds) were selected in the 27 Federative Units (FUs) by directed choice. To ensure the diversity of the sample, supporters with different sociodemographic characteristics (sex, age group, level of education) were selected.

Supporters sent the questionnaire link to 24 people from their social media, living in the same Federative Unit. The link was sent to an individual in each stratum composed of sex, age group (18–29; 30–44; 45–59; 60+) and level of education (some elementary school; elementary school/some high school; high school or higher).

The survey participants invited by supporters constituted the first wave of the recruitment chain. These participants, in turn, invited three to five people from their social media, composing the second wave of the recruitment chain. This process continued successively, ultimately composing the sample of individuals recruited in chain by social media. In the peer recruitment, it was recommended that only one person in each household should answer the questionnaire.

### Data analysis

In this article, the proportions of individuals in the ConVid-2 survey were estimated according to the categories of variables used in the post-stratification procedure of the data: sex (men; women), age group (18–39; 40–59; 60+ years), level of education (some high school or lower; high school or some college; college degree or higher), and race/skin color (white; nonwhite). These proportions were compared with those obtained from PNADC-2022.

For further investigation of the representativeness of the sample, some proportions of variables that were not used in the post-stratification procedure were analyzed. Among these variables are macro-regions, residence in some Brazilian capital, and some elementary school as level of education. These proportions were compared to PNADC-2022 estimates.

For the visualization of the scope of the ConVid-2 survey sample, a map of Brazil was drawn with dots, in which each dot represents a municipality with at least one participant in the sample. For illustrating the networks of recruiter-recruitee pairs, a figure of the recruitment networks obtained by the application of the RDS method was prepared.

Likewise, a comparison was made between the estimates of diagnosis of some noncommunicable chronic disease (NCD) and self-rated health obtained from ConVid-2 and those obtained from the 2019 National Survey of Health (*Pesquisa Nacional de Saúde* – PNS-2019)^27^.

Other health estimates were also compared to those of PNS-2019. Among them are behaviors such as current smoking, consumption of healthy and ultra-processed foods, consumption of alcoholic beverages at least once a week, and recommended practice of physical activity. PNS estimates are available at https://www.pns.icict.fiocruz.br/painel-de-indicadores-mobile-desktop/.

### Data availability statement

The dataset is not publicly available because it contains information that compromises the privacy of the survey participants.

## RESULTS

We analyzed 3,805 individuals who participated in the ConVid-2 survey from July to December 2023.

In Table 1, we present the distributions of the variables used for the post-stratification of the ConVid-2 data and the distributions of the same variables in PNADC-2022. We verified very similar proportions when comparing the distributions of the ConVid-2 variables with those of PNADC. In turn, the confidence intervals of the proportions estimated in ConVid-2 are much wider than those estimated in PNADC due to the size of the ConVid-2 sample, which is much smaller than that of PNADC.

**Table 1:** Comparison of the distributions of variables used for the post-stratification of data from ConVid2 – Behavior Survey with the distributions of variables from the 2022 Continuous National Household Sample Survey. Brazil, 2023.

Variables		ConVid-2			PNADC-2022	
		n	%	95%CI	%	95%CI
Sex						
	Men	1,787	47.0	41.5–52.5	48.1	47.9–53.1
	Women	1,974	51.9	47.1–56.7	51.9	51.7–57.3
	Other	44	1.1	0.4–3.4	–	–
Race/skin color						
	White	1,651	43.4	39.2–47.7	43.4	42.9–48.3
	Nonwhite	2,154	56.6	52.3–60.8	56.6	56.1–62.8
Level of education						
	Some high school or lower	1,692	44.5	35.4–54.0	44.5	44.0–49.4
	High school or some college	1,452	38.1	32.3–44.3	38.2	37.8–42.3
	College degree or higher	661	17.4	13.6–21.9	17.3	16.9–19.6
Age group (years)						
	18 to 39	1,746	45.9	38.4–53.6	45.9	45.5–50.8
	40 to 59	1,295	34.0	30.3–38.0	34.0	33.8–37.7
	60 or over	764	20.1	14.7–26.8	20.1	19.8–22.4

PNADC-2022: Behavior Survey with the distributions of variables in the 2022 Continuous National Household Sample Survey; CI: confidence interval.

**Figure 1:**
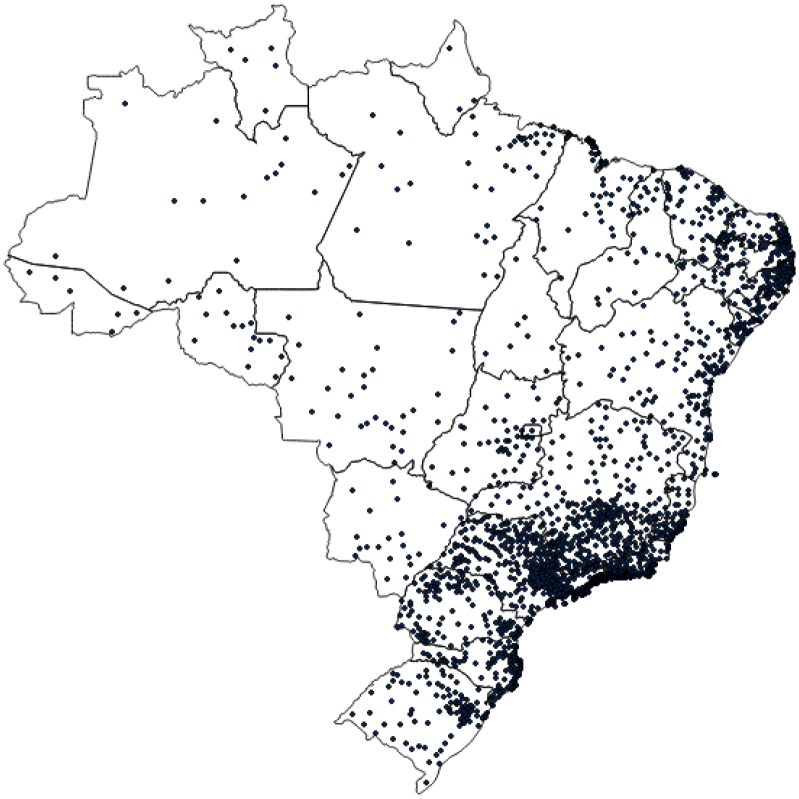
Distribution of municipalities with at least one respondent of the ConVid2 – Behavior Survey. Brazil, 2023.

In Figure 1, it is possible to see the scope of the ConVid-2 survey in Brazil. We can observe that all FUs have at least one participating municipality. The lowest number of respondents was recorded in the state of Amapá (seven people) and the highest in the state of Pernambuco (711), followed by Minas Gerais (555), Rio de Janeiro (406), and São Paulo (278).

**Figure 2:**
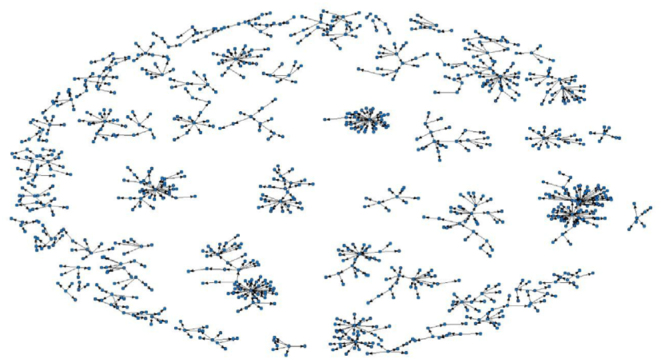
Example of network formation in the recruitment process of the Respondent-Driven Sampling method of a ConVid2 – Behavior Survey subsample. Brazil, 2023.

In Figure 2, we present the composition of networks in the recruitment process of the RDS method of a subsample of the ConVid-2 survey and concluded that, overall, the networks developed well.

In Table 2, we show the prevalence estimates of indicators that were not included in the post-stratification procedure. Regarding distribution by region, we found proportions of 8.1, 35.0, 36.2, 12.5, and 8.2% in the North, Northeast, Southeast, South, and Midwest regions, respectively. We observed overestimation of residents in the Northeast region and underestimation in the Southeast region in relation to those obtained in the PNADC-2022. As for the proportion of people who have some elementary school as level of education, the estimate of ConVid-2 was 21.6%, lower than that of PNADC-2022 (30.5%). Concerning the place of residence of the ConVid-2 participants, 42.8% lived in the state capital, with an estimated proportion almost twice (1.7) greater than that of PNADC-2022.

**Table 2:** Comparison of the distributions of selected variables that were not included in the post-stratification procedure of the ConVid2 – Behavior Survey data with the distributions of the variables from the 2022 Continuous National Household Sample Survey. Brazil, 2023.

Variables		ConVid-2			PNADC	
		n	%	95%CI	%	95%CI
Region						
	North	297	8.1	5.0–13.0	8.1	7.9–8.4
	Northeast	1,283	35.0	23.7–48.2	26.3	25.9–26.8
	Southeast	1,329	36.2	27.1–46.4	43.3	42.8–43.9
	South	458	12.5	4.8–28.8	14.6	14.2–14.9
	Midwest	303	8.2	4.7–14.1	7.7	7.5–7.9
Residence location						
	Capital	1,629	42.8	35.7–50.2	24.8	24.4–25.3
	Location other than the capital	2,176	57.2	49.8–64.3	75.2	74.7–75.6
Level of education						
	Some elementary school	820	21.6	15.9–28.6	30.5	30.1–30.8
	Elementary school or some high school	872	22.9	18.6–27.8	14.0	13.8–14.3
	High school or some college	1,452	38.2	32.3–44.3	38.2	37.8–38.5
	College degree or higher	661	17.4	13.6–21.9	17.4	16.9–17.9

PNADC-2022: Behavior Survey with the distributions of variables in the 2022 Continuous National Household Sample Survey; CI: confidence interval.

As shown in Table 3, the prevalence of NCDs estimated in the ConVid-2 survey is consistent with those found in PNS-2019 for most diseases. We highlight the highest prevalence of asthma, 8.5% in ConVid-2 and 5.3% in PNS-2019, and depression, 14.7% and 10.2%, respectively. Likewise, prevalence of at least one NCD was higher in ConVid-2 (55.4%) than in PNS-2019 (50.8%). Regarding self-rated health, there was a worsening in the post-pandemic period. The prevalence of good self-rated health decreased from 66.1%, according to PNS-2019, to 62.0%, according to ConVid-2.

**Table 3:** Prevalence (%) of self-reported diagnosis of chronic noncommunicable diseases, self-rated health, and health behaviors according to data from ConVid-2 and the National Survey of Health. Brazil, 2023.

Variables		ConVid-2		PNS-2019	
		%	95%CI	%	95%CI
NCDs					
	Hypertension	22.0	17.8–26.9	23.9	23.4–24.4
	Diabetes	8.9	6.9–11.4	7.7	7.4–8.0
	Heart disease	4.3	2.8–6.7	5.3	5.0–5.6
	Asthma	8.5	6.9–10.6	5.3	5.0–5.6
	Cancer	2.4	1.5–3.9	2.6	2.4–2.7
	Depression	14.7	12.6–17.1	10.2	9.9–10.6
	At least one NCD	55.4	51.5–59.4	50.8	50.1–51.4
Self-rated health					
	Good	62.0	57.5–66.2	66.1	65.5–66.7
	Fair	33.2	29.4–37.3	28.1	27.6–28.6
	Poor	4.8	3.2–7.1	5.8	5.5–6.0
Health behaviors					
	Current smoking	12.1	9.6–15.1	12.6	12.2–13.0
	Alcohol consumption (once a week or more)	21.9	18.6–25.6	26.4	25.8–27.0
	Leisure-time physical activity (recommended time)	32.7	28.6–37.2	30.1	29.4–30.7
	Screen use for three hours or more (leisure)	46.1	41.1–51.2	22.2	21.6–22.8
	Consumption of some fruit the day before the survey	65.8	62.6–68.9	67.3	66.7–67.9
	Consumption of some vegetable the day before the survey	66.8	63.2–70.2	70.9	70.4–71.5
	Consumption of ultra-processed foods the day before the survey	73.1	68.9–76.9	72.0	71.5–72.6

NCDs: chronic noncommunicable diseases; NHS: National Survey of Health; CI: confidence interval.

As for lifestyle habits, the percentages of current smoking, adequate leisure-time physical activity, consumption of healthy and unhealthy foods were close in both studies. Proportions of alcohol consumption at least once a week were lower in ConVid-2 (21.9%) than in PNS-2019 (26.4%). However, the greatest difference was observed in screen use after the COVID-19 pandemic, more than double the proportion found in PNS-2019 (Table 3).

## DISCUSSION

Unlike the first stage of the ConVid survey, carried out during the coronavirus pandemic by the snowball method^14^, in the ConVid-2 survey, the virtual RDS sampling technique was used, which has a well-established methodology for statistical data analysis^26^.

The use of the RDS method in the ConVid-2 survey represented a methodological advance regarding online surveys, as it can be considered a method with complex sampling design. This enables estimating the prevalence and respective confidence intervals of the indicators of interest and the performance of hypothesis tests^26^. In addition, the number of invitations for each participant is limited in the RDS method, conferring greater diversity to the sample and reducing the homophily effects^25^.

To give representativeness to the sample, the data were weighted by post-stratification procedures. These procedures were based on the population distribution by sex, age group, race/skin color, and level of education estimates of the 2022 PNADC^3^. It is worth noting that the proportions of people in each stratum found in ConVid-2 and PNADC were very similar, i.e., we may say that the sample is representative of the Brazilian population, at least in relation to the considered strata.

Regarding the indicators that were not part of the post-stratification procedure, such as geographic distribution, although the proportion of people in the Southeast region was underestimated and that of the Northeast region was overestimated, the research involved participants from the five Brazilian regions comprehensively. Seed stratification by FUs, as performed in ConVid-2, was certainly an important factor in achieving regional representativeness in the sample.

In the present study, we only used three categories of level of education in the post-stratification: some high school, high school and some college, and college degree or higher. The "some elementary school" category was not included in the post-stratification procedure. Although the proportion of people with some elementary school estimated in PNADC-2022 (30.5%) was 1.4 times greater than in ConVid-2 (21.6%), there was good representation of individuals with low levels of education. This finding represents an advance in relation to the first survey conducted in 2020^14^, possibly due to the greater availability of Internet access in the country^3^.

Regarding NCDs, the comparison with PNS-2019^27^ showed consistent results in the prevalence of hypertension, diabetes, cancer, and heart diseases. Conversely, we observed discrepancies in the prevalence of asthma and depression estimated in ConVid-2. As for asthma and depression, there may have been increases in the post-pandemic period. According to results from international studies, there was an increase in the prevalence of asthma after SARS-CoV-2 infection^28,29^.

Moreover, associations with long COVID were found, defined by the presence of symptoms and sequelae that persisted for three months or more, with the onset of some NCD. This would be a probable explanation for the increase in the prevalence of chronic diseases^30^. Persistence of COVID-19 symptoms and sequelae has also been associated with loss of quality of life, worsening of self-rated health, and mental health problems such as increased depression^31^.

Regarding health behaviors, we highlight the use of computers, tablets, or mobile phones for three hours or over. This was the only indicator that showed a considerable increase in prevalence and time of use after the COVID-19 pandemic. Authors of national^32^ and international^33^ studies have shown growth in screen use, demonstrating that the increase indeed occurred and does not constitute a divergence of results between ConVid-2 and PNS-2019.

As for lifestyle changes, the similarity found in most health behaviors in ConVid-2, compared to the same indicators estimated in PNS-2019, is noteworthy. On the one hand, there were no significant changes in unhealthy habits in the post-pandemic period when compared to the findings in 2019, before the pandemic onset, except for screen use and sedentary behavior. On the other hand, the results concerning smoking habit, leisure-time physical activity, and unhealthy food consumption were stable.

These findings allow us to infer that there were no advances and neither increases, as occurred during the pandemic^21,22^. Authors of a study on indicators of the Surveillance System for Risk and Protective Factors for Chronic Diseases by Telephone Survey (*Sistema de Vigilância de Fatores de Risco e Proteção para Doenças Crônicas por Inquérito Telefônico* – Vigitel) in the period 2006–2021 also showed that several indicators remained stable. Among them are the prevalence of smokers, regular and recommended consumption of fruits and vegetables as well as the consumption of ultra-processed foods^34^.

As regards the methodology used in the survey, the use of RDS and the inclusion of recruiter-recruitee pairs brought important benefits. This approach allowed us to estimate prevalence values and variances of the indicators of interest with greater reliability.

According to the stability in the protective and risk factors found in the comparison of the prevalence of health behaviors obtained from ConVid-2 and PNS-2019, there is absence of relevant progress in health promotion after the pandemic, which can be considered a setback in the control of NCDs. These findings reinforce the need for public policies aimed at adopting healthy behaviors and controlling NCDs in the post-pandemic scenario.

### Study limitations

Among the main limitations, we highlight the following: people without access to the Internet are unlikely to be selected; individuals with low levels of education often have difficulties filling out the online questionnaire, being underrepresented; due to peer recruitment by virtual RDS, participants are volunteers and the nonresponse rate, as well as selection odds, cannot be estimated.

Furthermore, to obtain a representative sample of the Brazilian population, a post-stratification procedure was used based on population estimates of PNADC-2022. However, the exclusion of a variable associated with one of the outcomes considered in the post-stratification procedure may cause errors in the average estimates of prevalence. As per the results, the sample has underrepresentation of people with some elementary school as level of education and overrepresentation of residents of Brazilian capitals. However, the comparison of the prevalence of chronic diseases and the health behaviors estimated in ConVid-2 showed consistency in the results, corroborating the consistency of the estimates compared to those of national face-to-face surveys.
