# Supplementary material for: Application of the Respondent-Driven Sampling method in an online survey in Brazil: COVID-19 morbidity in the post-pandemic period
Source: Rev Bras Epidemiol. 2026 Jan 30;29:e260004. doi: 10.1590/1980-549720260004 (PMC12858088; doi:10.1590/1980-549720260004)
Supplement: Translation

# Translation: Aplicação do método *Respondent-Driven Sampling* (RDS) em inquérito online no Brasil: análise da morbidade por COVID-19 no período pós-pandêmico

**DOI:** 10.1590/1980-549720260004.2

**Authors:** Paulo Roberto Borges de Souza, Giseli Nogueira Damacena, Euclides Ayres de Castilho, Wanessa da Silva Almeida, Crizian Saar Gomes, Deborah Carvalho Malta, Célia Landmann Szwarcwald

**Affiliations:** I Fundação Oswaldo Cruz, Instituto de Comunicação e Informação Científica e Tecnológica em Saúde, Laboratório de Informação em Saúde – Rio de Janeiro (RJ), Brasil.; II Universidade de São Paulo, Faculdade de Medicina, Departamento de Medicina Preventiva – São Paulo (SP), Brasil.; III Universidade Federal de Minas Gerais, Escola de Enfermagem – Belo Horizonte (MG), Brasil.

**Keywords:** COVID-19, Inquérito, Respondent-driven sampling, Pós-estratificação, Fatores sociodemográficos

## Abstract

**Objetivo:**

Descrever os procedimentos metodológicos utilizados para a coleta e análise de dados em inquérito *online* realizado no período de julho a dezembro de 2023 e verificar a se a amostra alcançada na pesquisa é representativa da população brasileira.

**Métodos:**

Estudo epidemiológico de corte transversal realizado pelas redes sociais virtuais, utilizando-se a metodologia *Respondent-Driven Sampling* (RDS) para a coleta de informações na população adulta brasileira. Para a ponderação da amostra, utilizou-se um procedimento de pós-estratificação com base nos dados sociodemográficos da Pesquisa Nacional de Amostra por Domicílios 2022 (PNADC-2022). As prevalências de doenças crônicas não transmissíveis (DCNT), autoavaliação de saúde e comportamentos de saúde estimadas na ConVid-2 foram comparadas às estimativas da Pesquisa Nacional de Saúde, 2019.

**Resultados:**

O total de 3.805 indivíduos participou da pesquisa ConVid-2. A comparação das variáveis sociodemográficas da ConVid-2 com as da PNADC mostrou proporções muito semelhantes nas duas pesquisas. Quanto às DCNT, a comparação com a PNS-2019 mostrou discrepâncias apenas nas prevalências de asma e depressão. Em relação aos hábitos, os percentuais de fumo atual, atividade física adequada no lazer e consumo de alimentos saudáveis e não saudáveis foram próximos nas duas pesquisas. A maior diferença ocorreu no uso de telas após a pandemia de COVID-19.

**Conclusão:**

O uso do método RDS com o registro dos pares recrutador-recrutado possibilitou obter estimativas mais fidedignas. As mudanças nos estilos de vida salientam a necessidade de ações voltadas à promoção de comportamentos saudáveis e ao controle das DCNT.

## INTRODUÇÃO

A grande expansão da *internet* junto com o crescimento e aprimoramento das tecnologias de informação tornaram a comunicação à distância muito mais abrangente e acessível às informações de saúde^1,2^.

No Brasil, em 2024, segundo os dados do Instituto Brasileiro de Geografia e Estatística (IBGE), 93,6% dos domicílios particulares permanentes utilizavam a *internet* ^3^. Diante deste acesso quase universal, ocorreram mudanças significativas na forma como os brasileiros se comunicam, obtêm e compartilham informações, compondo uma enorme rede de pessoas conectadas, independentemente de sua localização geográfica^4,5^.

As restrições impostas pela pandemia de coronavírus limitaram a realização de pesquisas presenciais. Os avanços tecnológicos na captação e no compartilhamento de informações em saúde e a necessidade de ampliar o conhecimento sobre a COVID-19 impulsionaram o uso de inquéritos *online* em diversos países^6^. Além da aderência às medidas de proteção e de restrição de contatos físicos^7^, os estudos abordaram outras questões relacionadas ao período de pandemia, como sintomas^8,9^, transtornos psicológicos^10,11^, dificuldades de acesso aos serviços de saúde^12^ e mudanças no estilo de vida^13^.

No Brasil, no ano de 2020, com a finalidade de investigar as mudanças no estilo de vida e nas condições de saúde da população brasileira durante a pandemia de COVID-19, foi realizada a ConVid — Pesquisa de Comportamentos em ambiente virtual^14^. Esta pesquisa utilizou um procedimento de amostragem em cadeia "bola de neve" para a coleta das informações^15^. Para adaptação do método ao ambiente virtual, o processo de recrutamento iniciou-se com o envio do *link* de acesso a um questionário eletrônico por *e-mail* ou por alguma rede social virtual. Os participantes receberam uma solicitação para compartilharem o convite de participação com sua rede de contatos^16^.

Os resultados da ConVid realizada na população adulta brasileira mostraram perdas importantes no trabalho e na renda, acentuando as desigualdades sociais^17^. Houve piora na percepção da saúde^18^, problemas no sono e nos transtornos psicológicos^19,20^, além de mudanças no estilo de vida. Destacam-se o aumento da inatividade física, do excesso de tempo de telas (celular, *tablet*, computador), do consumo de cigarros e do uso de bebidas alcoólicas^21,22^.

Em 2023, para investigar questões relacionadas à morbidade por COVID-19 no período pós-pandêmico, foi realizado novo inquérito denominado de "ConVid-2 — Pesquisa de Comportamentos". Utilizou-se um questionário eletrônico, autopreenchido pelo participante por meio de celular ou computador com acesso à *internet*. Nesta segunda etapa da pesquisa, foi feito um aprimoramento da metodologia de amostragem. O recrutamento foi realizado pelo método de amostragem *Respondent-Driven Sampling* (RDS)^23^, que se iniciou com o envio de um número fixo de convites com o *link* de acesso ao questionário eletrônico.

Os objetivos do presente artigo são os de descrever os procedimentos metodológicos utilizados para a coleta e análise de dados na ConVid-2 e verificar se a amostra alcançada na pesquisa é representativa da população brasileira.

## MÉTODOS

### Desenho do estudo

Estudo epidemiológico de corte transversal realizado por meio de redes sociais virtuais, utilizando a metodologia RDS para a coleta das informações na população adulta brasileira (18 anos ou mais) no período de julho a dezembro de 2023.

O projeto "ConVid-2 — Pesquisa de Comportamentos" foi aprovado pela Comissão Nacional de Ética em Pesquisa (CONEP) em 22 de dezembro de 2022, sob o número de protocolo 5.836.202.

A pesquisa foi realizada pela Fundação Oswaldo Cruz, em parceria com a Universidade Federal de Minas Gerais, a Universidade Estadual de Campinas, a Universidade Federal de Ouro Preto e a Universidade Federal de Sergipe.

Participaram do estudo pessoas com 18 anos ou mais de idade, residentes no Brasil durante a pandemia de COVID-19.

### Método *Respondent-Driven Sampling*

O método de recrutamento dos indivíduos foi o RDS, proposto em 1997 por Douglas Heckathorn^23^. Trata-se de uma variante do método de amostragem em cadeia, no qual os membros do grupo populacional em estudo recrutam seus pares para participar.

Ao aplicar o método RDS, os dados são coletados por meio de ciclos sucessivos de recrutamento, chamados de "ondas". Primeiramente, indivíduos da população alvo, denominados "sementes", são selecionados de forma não aleatória para participar do estudo, iniciando o processo de recrutamento. Solicita-se que as sementes recrutem um número fixo de indivíduos entre seus conhecidos do mesmo subgrupo populacional. É feito o registro das conexões dos pares recrutador-recrutado com base em códigos atribuídos a cada participante^24^.

No recrutamento pelo método RDS, a memória de recrutamento é dada onda por onda, obedecendo a um processo de Markov. Isso significa que as características do indivíduo recrutado dependem apenas daquelas do seu recrutador e não do recrutador do seu recrutador ou de participantes de ondas anteriores. Após suficiente número de ondas, as características dos indivíduos na amostra final tornam-se independentes daquelas da amostra inicial^24^.

A tendência de um participante a recrutar indivíduos com características semelhantes é chamada de "efeito homofilia"^25^. Como o registro das conexões dos pares recrutador-recrutado é preconizado no método RDS, o viés relacionado à seleção não aleatória de indivíduos é levado em consideração bem como a possível super-representação de certas características na população^25^. Assim, o RDS pode ser considerado um método de amostragem complexo, com probabilidades desiguais de seleção e conglomerados compostos de pessoas recrutadas pelo mesmo participantes. Para a estimação das variâncias dos indicadores de interesse, é utilizado o procedimento de *bootstrap* por meio de várias simulações de amostras geradas com o mesmo processo que originou a amostra total^26^.

### Tamanho da amostra

Sob o pressuposto de uma amostra aleatória simples, o tamanho de amostra mínimo necessário para estimar prevalências de 5% com intervalos de confiança de 95% e erro bilateral de 1% é de, aproximadamente, 1.800 pessoas. Uma vez que o recrutamento foi realizado por pares, considerou-se um efeito de desenho de 2 e o tamanho mínimo de amostra foi calculado em 3.600 pessoas^24^.

### Ponderação da amostra

Para obter uma amostra representativa da população de acordo com as características sociodemográficas da população brasileira de 18 anos ou mais, utilizou-se um procedimento de pós-estratificação^16^. A variáveis utilizadas foram: sexo (masculino; feminino), faixa etária (18–39; 40–59; 60+ anos), grau de escolaridade (ensino médio incompleto ou menos; ensino médio completo ou superior incompleto; superior completo ou mais) e raça/cor da pele (branca; não branca). O procedimento de pós-estratificação baseou-se nas estimativas populacionais da Pesquisa Nacional por Amostra de Domicílios Contínua, IBGE, 2022 (PNADC-2022)^3^.

### Coleta das informações

Para iniciar a etapa de coleta de informações por RDS, foram selecionados apoiadores (sementes) nas 27 Unidades da Federação (UF) por escolha dirigida. Para garantir a diversidade da amostra, foram selecionados apoiadores com diferentes características sociodemográficas (sexo, faixa de idade, grau de escolaridade).

Os apoiadores enviaram o *link* da pesquisa para 24 pessoas das suas redes sociais, residentes na mesma Unidade da Federação. O *link* foi enviado para um indivíduo em cada estrato composto de sexo, faixa de idade (18–29; 30–44; 45–59; 60+) e grau de escolaridade (ensino fundamental incompleto; ensino fundamental completo/ensino médio incompleto; ensino médio completo ou mais).

Os participantes da pesquisa convidados pelos apoiadores constituíram a primeira onda da cadeia de recrutamento. Eles, por sua vez, convidaram de três a cinco pessoas das suas redes sociais, compondo a segunda onda da cadeia de recrutamento. Esse processo continuou sucessivamente, formando ao fim a amostra de indivíduos recrutados em cadeia pelas redes sociais virtuais. No processo de recrutamento por pares, foi recomendado que apenas uma pessoa em cada domicílio respondesse ao questionário.

### Análise dos dados

Neste artigo, foram estimadas as proporções de indivíduos na pesquisa ConVid-2 segundo as categorias das variáveis utilizadas no procedimento de pós-estratificação dos dados: sexo (masculino; feminino), faixa etária (18–39; 40–59; 60+ anos), grau de escolaridade (ensino médio incompleto ou menos; ensino médio completo ou superior incompleto; superior completo ou mais) e raça/cor da pele (branca; não branca). Essas proporções foram comparadas com as obtidas na PNADC-2022.

Para investigação complementar da representatividade da amostra, foram analisadas algumas proporções de variáveis que não foram usadas no procedimento de pós-estratificação. Entre essas variáveis estão grande região, residência em alguma capital e ensino fundamental incompleto. Essas proporções foram comparadas às estimativas da PNADC-2022.

Para a visualização do alcance da amostra da pesquisa ConVid-2, elaborou-se um mapa do Brasil por pontos, em que cada ponto representa um município com pelo menos um participante da amostra. Para a exemplificação das redes dos pares recrutador-recrutados, foi apresentada uma figura das redes de recrutamento obtidas pela aplicação do método RDS.

Foi realizada, igualmente, a comparação das estimativas de diagnóstico de alguma doença crônica não transmissível (DCNT) e da autoavaliação de saúde obtidas na ConVid-2 com as obtidas na Pesquisa Nacional de Saúde, 2019 (PNS-2019) (27).

Outras estimativas de saúde também foram comparadas às da PNS-2019. Entre elas estão comportamentos como fumo atual, consumo de alimentos saudáveis e ultraprocessados, consumo de bebida alcoólica pelo menos uma vez na semana e prática recomendada de atividade física. As estimativas da PNS estão disponíveis em https://www.pns.icict.fiocruz.br/painel-de-indicadores-mobile-desktop/.

### Declaração de disponibilidade de dados

O conjunto de dados não está publicamente disponível devido conter informação que compromete a privacidade dos participantes da pesquisa.

## RESULTADOS

Foram analisados 3.805 indivíduos que participaram da pesquisa ConVid-2 de julho a dezembro de 2023.

Na Tabela 1, apresentam-se as distribuições das variáveis utilizadas para a pós-estratificação dos dados da ConVid-2 e as distribuições das mesmas variáveis na PNADC-2022. A comparação das distribuições das variáveis da ConVid-2 com as da PNADC mostra proporções muito semelhantes nas duas pesquisas. Já os intervalos de confiança das proporções estimadas na ConVid-2 são bem mais amplos do que os estimados na PNADC em razão do tamanho da amostra da ConVid-2, muito menor do que o da PNADC.

**Tabela 1:** Comparação das distribuições das variáveis utilizadas para a pós-estratificação dos dados da ConVid-2 — Pesquisa de Comportamentos com as distribuições das variáveis na Pesquisa Nacional por Amostra de Domicílios Contínua de 2022. Brasil, 2023.

Variáveis		ConVid-2			PNADC-2022	
		n	%	IC95%	%	IC95%
Sexo						
	Masculino	1.787	47,0	41,5–52,5	48,1	47,9–53,1
	Feminino	1.974	51,9	47,1–56,7	51,9	51,7–57,3
	Outro	44	1,1	0,4–3,4	-	-
Raça/Cor						
	Branca	1.651	43,4	39,2–47,7	43,4	42,9–48,3
	Não branca	2.154	56,6	52,3–60,8	56,6	56,1–62,8
Escolaridade						
	Médio incompleto ou menos	1.692	44,5	35,4–54,0	44,5	44,0–49,4
	Médio completo ou superior incompleto	1.452	38,1	32,3–44,3	38,2	37,8–42,3
	Superior completo ou mais	661	17,4	13,6–21,9	17,3	16,9–19,6
Faixa etária (anos)						
	18 a 39	1.746	45,9	38,4–53,6	45,9	45,5–50,8
	40 a 59	1.295	34,0	30,3–38,0	34,0	33,8–37,7
	60 ou mais	764	20,1	14,7–26,8	20,1	19,8–22,4

PNADC-2022: Pesquisa de Comportamentos com as distribuições das variáveis na Pesquisa Nacional por Amostra de Domicílios Contínua de 2022; IC: intervalo de confiança.

Na Figura 1, é possível visualizar o alcance da pesquisa ConVid-2 no Brasil. Observa-se que todas as UF têm pelo menos um município participante. O menor número de respondentes ocorreu no Amapá (sete pessoas) e o maior no estado de Pernambuco (711), seguido de Minas Gerais (555), Rio de Janeiro (406) e SP (278).

**Figura 1:** Distribuição dos municípios com pelo menos um respondente da ConVid-2 Pesquisa de Comportamentos. Brasil, 2023.

Na Figura 2, é apresentada a composição de redes no processo de recrutamento do método RDS de uma subamostra da ConVid-2 Pesquisa de Comportamentos, permitindo dizer que, de maneira geral, houve bom desenvolvimento das redes.

**Figura 2:** Exemplificação da formação de redes no processo de recrutamento do método *Respondent-Driven Sampling* de uma subamostra da ConVid-2 — Pesquisa de Comportamentos. Brasil, 2023.

Na Tabela 2, são apresentadas as prevalências de indicadores que não foram incluídos no procedimento de pós-estratificação. Com relação à distribuição por região, foram encontradas proporções de 8,1, 35,0, 36,2, 12,5, e 8,2% nas Regiões Norte, Nordeste, Sudeste, Sul e Centro-Oeste, respectivamente. Constatou-se a superestimação de moradores na Região Nordeste e subestimação na Região Sudeste em relação às obtidas na PNADC-2022. No que se refere à proporção de pessoas que têm ensino fundamental incompleto, a estimativa da ConVid-2 foi de 21,6%, menor do que a da PNADC-2022 (30,5%). Quanto ao local de residência dos participantes na ConVid-2, 42,8% moravam na capital do estado, com proporção estimada quase duas vezes (1,7) maior do que a da PNADC-2022.

**Tabela 2:** Comparação das distribuições de variáveis selecionadas que não participaram do procedimento de pós-estratificação dos dados da ConVid-2 — Pesquisa de Comportamentos com as distribuições das variáveis na Pesquisa Nacional por Amostra de Domicílios Contínua de 2022. Brasil, 2023.

Variáveis		ConVid-2			PNADC	
		n	%	IC95%	%	IC95%
Região						
	Norte	297	8,1	5,0–13,0	8,1	7,9–8,4
	Nordeste	1.283	35,0	23,7–48,2	26,3	25,9–26,8
	Sudeste	1.329	36,2	27,1–46,4	43,3	42,8–43,9
	Sul	458	12,5	4,8–28,8	14,6	14,2–14,9
	Centro-Oeste	303	8,2	4,7–14,1	7,7	7,5–7,9
Lugar de residência						
	Capital	1.629	42,8	35,7–50,2	24,8	24,4–25,3
	Não capital	2.176	57,2	49,8–64,3	75,2	74,7–75,6
Escolaridade						
	Fundamental incompleto	820	21,6	15,9–28,6	30,5	30,1–30,8
	Fundamental completo ou médio incompleto	872	22,9	18,6–27,8	14,0	13,8–14,3
	Médio completo ou superior incompleto	1.452	38,2	32,3–44,3	38,2	37,8–38,5
	Superior completo ou mais	661	17,4	13,6–21,9	17,4	16,9–17,9

PNADC-2022: Pesquisa de Comportamentos com as distribuições das variáveis na Pesquisa Nacional por Amostra de Domicílios Contínua de 2022; IC: intervalo de confiança.

Como apresentado na Tabela 3, as prevalências de DCNT estimadas na pesquisa ConVid-2 são coerentes com as encontradas na PNS-2019 para a maioria das doenças. Destacam-se as maiores prevalências de asma, de 8,5% na ConVid-2 e de 5,3% na PNS-2019, e de depressão, de 14,7 e 10,2%, respectivamente. Igualmente, a prevalência de pelo menos uma DCNT foi maior na ConVid-2 (55,4%) do que na PNS-2019 (50,8%). No que se refere à autoavaliação de saúde, houve piora no período pós-pandêmico. A prevalência encontrada de percepção boa da própria saúde reduziu de 66,1%, segundo a PNS- 2019, para 62,0%, de acordo com a ConVid-2.

**Tabela 3:** Prevalência (%) de diagnóstico autorreferido de doenças crônicas não transmissíveis, de autoavaliação da saúde e de comportamentos de saúde de acordo com os dados da ConVid-2 e da Pesquisa Nacional de Saúde. Brasil, 2023.

Variáveis		ConVid-2		PNS-2019	
		%	IC95%	%	IC95%
DCNT					
	Hipertensão arterial	22,0	17,8–26,9	23.9	23.4–24.4
	Diabetes	8,9	6,9–11,4	7.7	7,4–8,0
	Doença do coração	4,3	2,8–6,7	5,3	5,0–5,6
	Asma	8,5	6,9–10,6	5,3	5,0–5,6
	Câncer	2,4	1,5–3,9	2,6	2,4–2,7
	Depressão	14,7	12,6–17,1	10,2	9,9–10,6
	Pelo menos uma DCNT	55,4	51,5–59,4	50,8	50,1–51,4
Autoavaliação da saúde					
	Boa	62,0	57,5–66,2	66,1	65,5–66,7
	Regular	33,2	29,4–37,3	28,1	27,6–28,6
	Ruim	4,8	3,2–7,1	5,8	5,5–6,0
Comportamentos de saúde					
	Fumo atual	12,1	9,6–15,1	12,6	12,2–13,0
	Consumo de álcool (1 vez por semana ou mais)	21,9	18,6–25,6	26,4	25,8–27,0
	Atividade física no lazer (tempo recomendado)	32,7	28,6–37,2	30,1	29,4–30,7
	Uso de telas por 3 horas ou mais (lazer)	46,1	41,1–51,2	22,2	21,6–22,8
	Consumo de alguma fruta no dia anterior à pesquisa	65,8	62,6–68,9	67,3	66,7–67,9
	Consumo de alguma hortaliça no dia anterior à pesquisa	66,8	63,2–70,2	70,9	70,4–71,5
	Consumo de alimentos ultraprocessados no dia anterior à pesquisa	73,1	68,9–76,9	72,0	71,5–72,6

DCNT: doenças crônicas não transmissíveis; PNS: Pesquisa Nacional de Saúde; IC: intervalo de confiança.

Quanto aos hábitos, os percentuais de fumo atual, atividade física adequada no lazer, bem como os de consumo de alimentos saudáveis e não saudáveis foram próximos nas duas pesquisas. Proporções de consumo de álcool pelo menos uma vez na semana foram menores na ConVid-2 (21,9%) do que na PNS-2019 (26,4%). Entretanto, a maior diferença ocorreu no uso de telas após a pandemia de COVID-19, mais do que o dobro da proporção encontrada na PNS-2019 (Tabela 3).

## DISCUSSÃO

Diferentemente da primeira etapa da ConVid realizada durante a pandemia de coronavírus pelo método "bola de neve"^14^, na pesquisa ConVid-2 se utilizou a técnica de amostragem RDS virtual, que dispõe de metodologia consagrada de análise estatística dos dados^26^.

O uso do método RDS na pesquisa ConVid-2 representou um avanço metodológico no tocante aos inquéritos em ambiente virtual, pois pode ser considerado um método com desenho complexo de amostragem. Isso possibilita estimar as prevalências e respectivos intervalos de confiança dos indicadores de interesse e a realização de testes de hipóteses^26^. Além disso, o número de convidados para cada participante é limitado no método RDS, dando maior diversidade à amostra e reduzindo os efeitos de homofilia^25^.

Para dar representatividade à amostra, os dados foram ponderados por procedimentos de pós-estratificação. Esses procedimentos basearam-se na distribuição da população por sexo, faixa de idade, raça/cor e escolaridade estimativas da PNAD contínua do ano de 2022^3^. Nota-se que as proporções de pessoas em cada estrato encontradas na ConVid-2 e na PNADC foram muito semelhantes, ou seja, é possível dizer que amostra é representativa da população brasileira, pelo menos no que se refere aos estratos considerados.

Com relação aos indicadores que não fizeram parte do procedimento de pós-estratificação, como a distribuição geográfica, embora a proporção de pessoas na Região Sudeste tenha sido subestimada e a da Região Nordeste superestimada, a pesquisa envolveu participantes das cinco regiões brasileiras de forma abrangente. A estratificação de sementes por UF, como realizada na ConVid-2, foi certamente um fator importante para alcançar a representatividade regional na amostra.

Na presente pesquisa, foram utilizadas apenas três categorias de grau de escolaridade na pós-estratificação: nível médio incompleto, médio completo ou superior incompleto e superior completo ou mais. A categoria ensino fundamental incompleto não foi incluída no procedimento de pós-estratificação. Apesar da proporção de pessoas com ensino fundamental incompleto estimada na PNADC-2022 (30,5%) ser 1,4 vez maior do que na ConVid-2 (21,6%), houve boa representação dos indivíduos de baixa escolaridade. Esse resultado representa um avanço em relação à primeira pesquisa realizada em 2020 (14), possivelmente pela maior disponibilidade de acesso à *internet* no país^3^.

No que diz respeito às DCNT, a comparação com a PNS-2019^27^ mostrou resultados coerentes nas prevalências de hipertensão arterial, diabetes, câncer e doença do coração. Por outro lado, foram observadas discrepâncias nas prevalências de asma e depressão estimadas na ConVid-2. Tanto no caso da asma como da depressão, é possível que tenham ocorrido, de fato, aumentos no período pós-pandêmico. Resultados de estudos internacionais mostraram o aumento da prevalência de asma após a infecção por SARS-CoV-2^28,29^.

Adicionalmente, foram encontradas associações da COVID longa, definida pela presença de sintomas e sequelas que persistiram por três meses ou mais, com o desenvolvimento de alguma DCNT. Essa seria uma explicação provável para o aumento na prevalência de doenças crônicas^30^. A persistência dos sintomas e sequelas da COVID-19 tem sido associada, igualmente, à perda da qualidade de vida, à piora da autopercepção da saúde e a problemas de saúde mental, como o aumento da depressão^31^.

Quanto aos comportamentos de saúde, destaca-se o uso de computador, *tablet* ou telefone celular por 3 horas ou mais. Este foi o único indicador que mostrou grande aumento na prevalência e no tempo de uso após a pandemia de COVID-19. Estudos nacionais^32^ e internacionais^33^ têm mostrado crescimento no uso de telas, demonstrando que o aumento ocorreu de fato e não constitui uma divergência de resultados entre a ConVid-2 e a PNS-2019.

Com relação às mudanças no estilo de vida, a semelhança encontrada na maioria dos comportamentos de saúde na ConVid-2 em relação aos mesmos indicadores estimados na PNS-2019 revela aspectos importantes. Por um lado, não houve alterações significativas dos hábitos não saudáveis no período pós-pandêmico quando comparados aos achados em 2019, antes da chegada da pandemia. As exceções foram o uso de telas e o comportamento sedentário. Por outro lado, os resultados sobre o hábito de fumo, prática de atividade física no lazer e consumo de alimentos não saudáveis mostraram estabilidade.

Esses achados permitem inferir que não houve avanços, mas também não houve aumentos, como ocorreu durante a pandemia^21,22^. Estudo com indicadores do Sistema de Vigilância de Fatores de Risco e Proteção para Doenças Crônicas por Inquérito Telefônico (Vigitel) no período 2006–2021 mostrou, igualmente, que vários indicadores se mantiveram estáveis. Entre eles estão a prevalência de fumantes, o consumo regular e recomendado de frutas e hortaliças, bem como o consumo de alimentos ultraprocessados^34^.

Em conclusão, no que se refere à metodologia usada no inquérito, a utilização do RDS e a inclusão dos pares recrutador-recrutado trouxeram benefícios importantes. Essa abordagem possibilitou a estimação das prevalências e variâncias dos indicadores de interesse e a obtenção de estimativas com maior fidedignidade.

A estabilidade nos fatores de proteção e de risco encontrada na comparação das prevalências dos comportamentos de saúde obtidos na ConVid-2 e na PNS-2019 indica ausência de progressos relevantes na promoção da saúde após a pandemia e pode ser considerada um retrocesso no controle das DCNT. Esses achados reforçam a necessidade de políticas públicas voltadas à adoção de comportamentos saudáveis e ao controle das DCNT no cenário pós-pandêmico.

### Limitações

Entre as principais limitações, destacam-se: pessoas sem acesso à internet têm probabilidade zero de serem selecionadas; indivíduos de baixa escolaridade têm, frequentemente, dificuldades de preencher o questionário *online*, sendo sub-representados; por conta do recrutamento por pares pelo RDS virtual, os participantes são voluntários e a taxa de não resposta assim como as probabilidades de seleção não podem ser estimadas.

Adicionalmente, para a obtenção de uma amostra representativa da população brasileira, utilizou-se um procedimento de pós-estratificação baseado nas estimativas populacionais da PNADC-2022. Contudo, a exclusão de uma variável associada a um dos desfechos considerados no procedimento de pós-estratificação pode causar erros nas estimativas médias de prevalências. Como os resultados mostraram, a amostra tem sub-representação das pessoas com ensino fundamental incompleto e super-representação de residentes nas capitais do país. Todavia, a comparação das prevalências de doenças crônicas e dos comportamentos de saúde estimadas na ConVid-2 demonstrou coerência nos resultados, corroborando a consistência das estimativas em comparação com as de pesquisas nacionais presenciais.
